# MEYER’S DYSPLASIA IN THE DIFFERENTIAL DIAGNOSIS OF HIP PAIN IN CHILDHOOD

**DOI:** 10.1590/1413-785220243205e278967

**Published:** 2024-10-28

**Authors:** Fabio Carramão Narimatsu, Pedro Alcantara Barroso, Alceu José Fornari Gomes Chueire, Paulo Humberto Mem Martendal Costa, Miguel Akkari, Claudio Santili

**Affiliations:** 1. Irmandade da Santa Casa de Misericordia de São Paulo, Departamento de Ortopedia e Traumatologia “Pavilhao Fernandinho Simonsen”, Grupo de Ortopedia e Traumatologia, Sao Paulo, SP, Brazil.; 2. Hospital da Criança e Maternidade (HCM), Faculdade de Medicina de São Jose do Rio Preto, Grupo de Ortopedia Pediatrica, Sao Jose do Rio Preto, SP, Brazil.; 3. Irmandade da Santa Casa de Misericordia de São Paulo, Departamento de Ortopedia e Traumatologia “Pavilhão Fernandinho Simonsen”, Grupo de Ortopedia Pediátrica, Sao Paulo, SP, Brazil.; 4. Santa Casa de Misericordia de São Paulo, Faculdade de Ciências Médicas, Sao Paulo, SP, Brazil.

**Keywords:** Legg-Calve-Perthes Disease, Hip, Intermittent Claudication, Hip Joint, Doença de Legg-Calve-Perthes, Quadril, Claudicação Intermitente, Articulação do Quadril

## Abstract

Objective: This study reviews the literature and shares clinical experiences, emphasizing its diagnostic relevance in children under 5 years of age. Method: We examined 169 cases of Legg-Calvé-Perthes disease (LCPD) in patients in this age group. We analyzed medical records and images, observing variables such as age, gender, complaints, treatment, and outcomes. Results: We studied 20 patients with Meyer’s dysplasia, representing 1.4% of LCPD cases in children. The majority were boys (85%) with symptom onset at 38 months. Claudication (25%) and mild pain (40%) were the main complaints. Radiographic findings showed a smaller, granular, or asymmetric nucleus. The average follow-up was 6.4 years, with interventional treatment in 5 cases. Most showed complete reossification and centralization of the femoral head. Conclusion: Meyer’s dysplasia is a rare condition that affects the hip in children under 5 years of age, predominantly in boys. It usually does not require intensive treatment; clinical and radiological follow-up is sufficient. However, it is important to be aware of possible unfavorable progressions, requiring more aggressive treatment to prevent complications. ***Level of evidence III, Retrospective comparative study.***

## RESUMO

Objetivo: Este estudo objetiva revisar a literatura e compartilhar experiências clínicas, enfatizando sua relevância diagnóstica em crianças com menos de 5 anos. Método: Estudamos 169 casos de Doença de Legg-Calvé-Perthes (DLCP) em pacientes desta faixa etária. Analisamos prontuários e imagens, observando variáveis como idade, gênero, queixas, tratamento e resultados. Resultados: Estudamos 20 pacientes com displasia de Meyer, que representavam 1,4% dos casos de DLCP em crianças. A maioria eram meninos (85%) com início dos sintomas aos 38 meses. Claudicação (25%) e dor leve (40%) foram as principais queixas. Achados radiográficos mostraram núcleo menor, granular ou assimétrico. O acompanhamento médio foi de 6,4 anos, com tratamento intervencionista em cinco dos casos. A maioria apresentou reossificação completa e centralização da cabeça femoral. Conclusão: A Displasia de Meyer é uma condição rara que afeta o quadril em crianças menores de 5 anos, predominantemente em meninos. Geralmente, não requer tratamento intensivo; os acompanhamentos clínico e radiológico são suficientes. Contudo, é importante estar ciente de possíveis evoluções desfavoráveis, requerendo tratamento mais agressivo para evitar sequelas. ***Nível de Evidência III, Estudo restrospectivo comparativo.***

## INTRODUCTION

 Meyer’s dysplasia (MD) or “Dysplasia Epiphysealis Capitis Femoris” is a rare disorder that leads to a change in the proximal epiphyseal core of the femur. It was first described by Pedersen ^1^ in 1960 and later described in detail by Meyer ^2^ in 1964. It was based on observations in a group of patients with Legg-Calvé-Perthes disease (LCPD) with benign outcomes and younger age groups. 

 The patient may present with episodes of mild pain and limp as a defensive posture or remain asymptomatic. ^1^ ^-^ ^11^ On a plain radiograph, a reduced size of the affected epiphysis compared to the unaffected side may be observed with unilateral involvement, as described by Meyer, who described that ossification does not occur until about 2 years of age. When it happens, the small epiphyseal nucleus has a pathological appearance and irregular mineralization pattern resembling a “mulberry” or “flaky” appearance. ^2^

 Its clinical relevance lies in its importance as a differential diagnosis to other more serious hip disorders in preschool-aged children, such as infectious processes and especially LCPD. ^3^ ^-^ ^6^ Lack of awareness of its existence and evolutionary features for proper diagnostic clarification (of aetiology) can lead to unnecessary additional testing, hospitalization and surgery. ^5^ ^-^ ^11^

 In its natural course, Meyer’s dysplasia is benign in the vast majority of cases, with symptoms improving and no functional or structural impairment occurring in adulthood. However, some cases may take an atypical course leading to deformities of the femoral head. Therefore, it is important to better understand the development and characteristics of the disease and to identify factors that may lead to a different prognosis than usual. ^6^ ^-^ ^11^

We aimed to review the current literature on this condition, add our experience to its evolution, discuss its peculiarities and highlight the importance of its recognition in the differential diagnosis of hip pain in children under 5 years old.

## MATERIAL AND METHODS

The research project was submitted to the ReserachEthics Committee of the Brazil Platform and approved for implementation under CAAE number 39750814.0.0000.5479.

In this retrospective study, we analyzed the medical records and imaging examinations of patients who were first diagnosed with LCPD in the Department of Orthopedics and Traumatology of Santa Casa de São Paulo between 1978 and 2023. A total of 169 records were reviewed.

Inclusion criteria were records and radiographs of patients with clinical information and radiologic features compatible with the disease, in children under 5 years old with a diagnostic hypothesis of MD. Exclusion criteria were patients without documentation in the outpatient follow-up, such as lack of radiographs until reossification of the proximal femoral epiphyseal nucleus or skeletal maturity, as well as incomplete or missing clinical data.

The variables analyzed were age, gender, laterality, complaints or reason for seeking medical care, duration of development, age at onset of complaints, radiograph, treatment applied, and joint outcome.

## RESULTS

Twenty patients were diagnosed with Meyer’s dysplasia, affecting 27 hips out of a total of 1322 children diagnosed with LCPD, representing 1.4% of the total cases. Of these, seventeen (85%) were boys and only three (15%) were girls. Seven (35%) children had bilateral involvement. The average age at first consultation was 38 months (with a range of 26 to 48 months).

The reason for seeking medical attention was limping in 5 (25%) patients, 8 children (40%) had mild hip pain, 7 (35%) had both symptoms and in 1 case (5%) it was an X-ray finding after a traumatic event. The mean duration of symptoms was 7.2 months (1-24 months). The mean age at onset of symptoms was 32.7 months (23-45 months).

**Figure 1.**
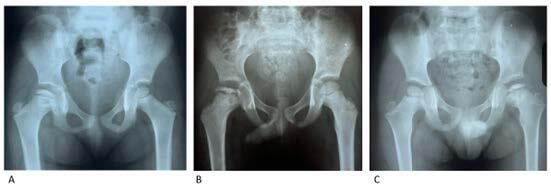
Radiographs of pelvis in anteroposterior orientation illustrating the patterns of involvement in Meyer’s epiphysitis - A – asymmetric pattern; B – granular pattern; C - mixed pattern.

 Regarding the radiographic findings, we found that in 7 children (35%) the nucleus was smaller in size and height than on the healthy side (characterized as an asymmetric pattern), in two (10%) the pattern was granular and in 6 (30%) both aspects, generally affecting the entire epiphyseal nucleus ( Figure 1 ). 

**Figure 2.**
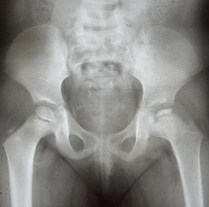
X-ray of the pelvis (anteroposterior view) at the initial examination at 3 years and 8 months old.

**Figure 3.**
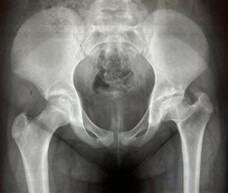
Radiograph of the pelvis (anteroposterior view) at the final examination at 11 years and 11 months.

 Follow-up ranged from 3 to 14 years with a mean of 6.4 years. Five patients underwent interventional treatment, with two patients receiving skin traction followed by a cast in mid-abduction and three patients receiving an abduction cast without prior traction. In the remaining 15 patients, no intervention was performed, but only outpatient follow-up care was provided. Radiological follow-up revealed complete reossification and centralization of the femoral head in all cases. One notable case among the patients examined concerned a female patient who had been treated in our clinic at the age of 3 years. The initial radiographs showed a compaction of the epiphyseal ossification nucleus ( Figure 2 ), which developed into a deformity of the femoral head during the follow-up examination ( Figure 3 ). However, she maintained a centralized articulation with non-spherical congruency and did not undergo corrective surgery up to the time of the study. 

## DISCUSSION

Meyer’s dysplasia is a rare disorder described by Pedersen in 1960 and later defined more precisely by Meyer in 1964. According to the observations of these authors, it is a disorder with features that differ from those observed in a group of patients originally diagnosed with LCPD.

 The symptoms of MD are usually milder, onset occurs at a younger age, usually under five years, and bilateral cases are more common. ^1^ ^-^ ^11^ When present, symptoms can range from complaints of limping, mild pain, changes in a sitting position, “waddling” gait” – external rotation and abduction - to asymptomatic cases found on examination for other reasons. ^4^ ^,^ ^6^ ^,^ ^7^ ^,^ ^8^ Pain, either isolated or associated with limping, was the most common reason for seeking medical attention and accounted for 40% of the cases examined in our series. Only one patient was identified as an X-ray finding in a child being examined for abdominal disease. 

 The etiology remains undetermined, there are various possibilities. A vascular cause has been proposed by Meyer due to ischemia as well as by Batory, who theorizes about a congenital vascular malformation. ^2^ ^,^ ^4^ However, since the advent and availability of investigations such as angiography, it is no longer possible to identify changes in the vascularization of the secondary epiphyseal nucleus of the proximal femur, which deviates from these hypotheses. ^3^ ^,^ ^4^ Because the disorder occurs in a bone development zone, an endocrinologic etiology has been proposed, ^4^ but we have found no conclusive studies on this etiology. 

 Other authors consider MD to be a precursor to LCPD, an earlier stage, ^2^ a theory that differs from the more accepted literature that distinguishes them as two distinct pathologies. ^3^ ^-^ ^11^ When the diagnoses overlap, the typical radiographic changes of MD initially occur without the typical LCPD changes such as subchondral bone fractures and lateral subluxation. Subsequently, these changes occur and merge with the MD changes, with extensive and massive necrosis noted. The well-established chronologic study supports the theory that these are different conditions and not stages of the same disease, ^4^ ^-^ ^9^ a theory with which we agree. 

 The radiographic features of MD can be described as changes in the bony nucleus with evidence of delayed ossification compared to the contralateral side, showing marked asymmetry in the size and height of this nucleus. ^2^ ^,^ ^6^ Another characteristic shape is the stippled and granular pattern described as “mulberry-like” or “golf ball”. ^2^ ^,^ ^4^

 In addition to the various radiologic presentations of MD, other types of changes have been found in the literature. Khermosh analyzed the distance between the upper edge of the metaphysis and the Hilgenreiner line and found a reduction (about 30-50% in his case series) on the affected side in unilateral cases. ^8^ Necrosis can occur in Meyer’s disease, as observed in our study, but more subtly than in LCPD, where there is extensive, homogeneous, and dense necrosis. Other features of LCPD that are not found in MD are lateral subluxation, subchondral fracture and changes in the femoral neck. ^2^ ^,^ ^6^ Other investigation methods such as scintigraphy show normal results and MRI shows only fragmentation and reduction of the epiphysis. ^3^ ^,^ ^4^ ^,^ ^8^ In our patients, the asymmetric pattern was most common, followed by the mixed type, where both asymmetry and granulation were present in the ossification. 

 Delayed ossification of the secondary nucleus of the proximal femur or even its “absence”, seen on radiographs of 2-year-old children, can occur in MD. This fact inspired theories that the etiology of MD is related to an ossification disorder rather than changes in blood supply and necrosis of the epiphyseal nucleus, as occurs in LCPD. ^4^ ^-^ ^6^ In MD, the nucleus would already be altered at the onset of ossification, whereas in LCPD this structure is completely normal before necrosis. ^2^ ^,^ ^4^ In MD, the epiphyseal nucleus would have an as-yet-unknown ability to normalize, favoring natural resolution. ^4^

 The delay in bone age has also been observed in MD by different authors, although the methodology was evaluated differently, making this observation difficult to compare. However, it remains a consistent finding in this disease. In the study by Xiao-Tang Sun, the method of Tanner and Whitehouse 3 (TW3) was applied and a decrease in bone age in the relationship between radius-ulna and other bones as well as the age of carpal bones in Meyer dysplasia was observed following the pattern of “delay and then recovery”. ^3^ Compared to LCPD, MD was noted to have a greater initial delay in bone maturation and a more pronounced decrease in the relationship between radius-ulna and other bones. However, in MD, bone age normalized at around five years of age, which was noted for both the carpal bones and the relationship between the radius and ulna and other bones, whereas in LCPD this normalization did not occur until around eight years of age. The finding that the delay in bone age resolves more rapidly in Meyer dysplasia is consistent with the milder course of the disease. ^3^

 The epidemiologic profile of the 20 patients enrolled in this study was similar to that found in the literature, with a higher incidence in males and an earlier onset of the disease compared to LCPD. The average age at diagnosis was slightly higher than reported in the literature, averaging 38 months, while other authors reported values between 24 and 36 months. ^4^ ^,^ ^8^ This can be justified by the duration of the complaints and the resulting delay in seeking medical care and clarification of the diagnosis, as the patients in this study reported that the clinical condition had manifested itself on average 7.2 months earlier, increasing the average age of onset to 32.7 months. Bilaterality, which varies between 42 and 59% ^2^ ^,^ ^6^ in the literature, was observed in 35% of the cases analyzed here. 

 In addition to LCPD, the differential diagnoses of MD can also include epiphyseal dysplasia, hypothyroidism and the consequences of infectious processes in the hip. ^3^ ^-^ ^11^

 The natural course of MD is usually benign and requires no treatment. Immobilization, plaster casts, orthoses and traction have been mentioned in some of the literature. Nevertheless, it is important to emphasize that there is a possibility of an unfavorable course that can lead to secondary conditions, chronic pain and joint restrictions. Therefore, appropriate treatment is essential to support patients with such a progression, ^4^ ^,^ ^10^ ^,^ ^11^ which sometimes requires corrective surgery. 

 Such conditions can be treated in the active phase with conservative strategies such as immobilization, offloading, skin or skeletal traction. In the chronic phase, surgical approaches such as osteotomies and even joint prostheses can be used to relieve pain, improve mobility or correct the deformities of secondary conditions. ^1^ ^-^ ^11^ The change in the shape of the femoral head may be characterized by a certain flattening or reduction of its residual volume, the coxa vara, and a widening of the femoral neck. ^3^ ^,^ ^4^ ^,^ ^7^ ^-^ ^11^ Appropriate follow-up after the initial resolution of the condition, even if benign, is essential to monitor for the development of sequelae or new symptoms. 

 Of the patients we studied, seven received a cast with the lower limbs in abduction, one received percutaneous traction and two others received traction followed by a cast in abduction. However, we believe that clinical follow-up and observation are sufficient in the vast majority of cases. ^3^ ^-^ ^6^ ^,^ ^8^ ^-^ ^11^ Reossification, with the disappearance of radiographic changes, occurs completely on average three years after the onset of the disease, unlike LCPD, which takes an average of 5-7 years. ^2^ ^,^ ^11^ The final result is generally a spherical and centralized hip joint with good results in terms of Stulberg parameters and concentric circles of Mose. ^6^ ^,^ ^12^ ^,^ ^13^ One of the cases studied had a more severe deformity but showed no symptoms or limitation of range of motion and did not require surgical treatment during the eight years of follow-up. Despite the generally good outcome, it is worth noting that follow-up of our patients and appropriate treatment may have prevented the worsening of sequelae and the resulting need for more invasive measures. 

## CONCLUSION

MD is a rare disease that affects the hip in children of younger age (under 5 years), occurs predominantly in males, has more bilateral involvement and a more favorable course compared to LCPD. The diagnosis is made based on clinical/epidemiologic aspects and simple radiology. Due to the favorable prognosis, more restrictive treatment or hospitalization is usually not required as clinical/orthopedic observation and follow-up are sufficient. However, the possibility of an unfavorable outcome must be taken into account, which requires rapid and efficient therapeutic intervention to prevent secondary damage and the need for more aggressive procedures.
